# Novel Versus Conventional Sequencing of β-Blockers, Sodium/Glucose Cotransportor 2 Inhibitors, Angiotensin Receptor-Neprilysin Inhibitors, and Mineralocorticoid Receptor Antagonists in Stable Patients With Heart Failure With Reduced Ejection Fraction (NovCon Sequencing Study): Protocol for a Randomized Controlled Trial

**DOI:** 10.2196/44027

**Published:** 2025-03-10

**Authors:** Sumanth Karamchand, Tsungai Chipamaunga, Poobalan Naidoo, Kiolan Naidoo, Virendra Rambiritch, Kevin Ho, Robert Chilton, Kyle McMahon, Rory Leisegang, Hellmuth Weich, Karim Hassan

**Affiliations:** 1 Department of Cardiology Faculty of Medicine and Health Sciences Stellenbosch University Stellenbosch South Africa; 2 Department of Internal Medicine Nelson R Mandela School of Medicine, King Edward VIII Hospital University of Kwa-Zulu Natal Durban South Africa; 3 Department of Medicine Nelson R Mandela School of Medicine, King Edward VIII Hospital University of Kwa-Zulu Natal Durban South Africa; 4 School of Law University of South Africa Pretoria South Africa; 5 Division of Pharmacology Department of Pharmaceutical Sciences University of Kwa-Zulu Natal Durban South Africa; 6 Department of Cardiology Life Flora Hospital Roodeport South Africa; 7 Division of Cardiology Department of Medicine University of Texas Health Science Center San Antonio, TX United States; 8 ABX-CRO advanced pharmaceutical services Dresden Germany; 9 Department of Pharmacy University of Uppsala Uppsala Sweden; 10 Division of Cardiology Faculty of Medicine and Health Sciences Tygerberg Hospital Stellenbosch South Africa; 11 Department of Cardiology Stellenbosch University Stellenbosch South Africa; 12 Department of Medicine Stellenbosch University Stellenbosch South Africa; 13 Department of Cardiology Life Bay View Private Hospital Mossel Bay South Africa

**Keywords:** heart failure, SGLT2i, sodium/glucose cotransporter 2 inhibitors, ARNi, angiotensin receptor-neprilysin inhibitors, HFrEF, heart failure with reduced ejection fraction, idiopathic dilated cardiomyopathy, heart, chronic heart failure, patient, control, clinical, adult, cardiomyopathy, therapy

## Abstract

**Background:**

Chronic heart failure has high morbidity and mortality, with approximately half of the patients dying within 5 years of diagnosis. Recent additions to the armamentarium of anti–heart failure therapies include angiotensin receptor-neprilysin inhibitors (ARNIs) and sodium/glucose cotransporter 2 inhibitors (SGLT2is). Both classes have demonstrated mortality and morbidity benefits. Although these new therapies have morbidity and mortality benefits, it is not known whether rapid initiation is beneficial when compared with the conventional, slower-stepped approach. Many clinicians have been taught that starting with low-dose therapies and gradually increasing the dose is a safe way of intensifying treatment regimens. Pharmacologically, it is rational to use a combination of drugs that target multiple pathological mechanisms, as there is potential synergism and better therapeutic outcomes. Theoretically, the quicker the right combinations are used, the more likely the beneficial effects will be experienced. However, rapid up-titration must be balanced with patient safety and tolerability.

**Objective:**

This study aims to determine if early addition of ARNIs, SGLT2is, β-blockers, and mineralocorticoid receptor antagonists (within 4 weeks), when compared with the same therapies initiated slower (within 6 months), will reduce all-cause mortality and hospitalizations for heart failure in patients with stable heart failure with reduced ejection fraction.

**Methods:**

This is a single-center, randomized controlled, double-arm, assessor-blinded, active control, and pragmatic clinical trial. Adults with stable heart failure with reduced ejection fraction and idiopathic dilated cardiomyopathy will be randomized to conventional sequencing (the control arm; over 6 months) of anti–heart failure therapies, and a second arm will receive rapid sequencing (over 4 weeks). Study participants will be followed for 5 years to assess the safety, efficacy, and tolerability of the 2 types of sequencing. Posttrial access and care will be provided to all study participants throughout their lifespan.

**Results:**

We are currently in the process of obtaining ethical clearance and funding.

**Conclusions:**

We envisage that this study will help support evidence-based medicine and inform clinical practice guidelines on the optimal rate of sequencing of anti–heart failure therapies. A third placebo arm was considered, but costs would be too much and not providing study participants with therapies with known morbidity and mortality benefits may be unethical, in our opinion. Given the post–COVID-19 economic downturn and posttrial access to interventions, a major challenge will be acquiring funding for this study.

**International Registered Report Identifier (IRRID):**

PRR1-10.2196/44027

## Introduction

### Background

The current management of heart failure with reduced ejection fraction (HFrEF) has been revolutionized by the emergence of novel classes of drugs, namely, angiotensin receptor-neprilysin inhibitors (ARNIs) and sodium/glucose cotransporter 2 inhibitors (SGLT2is) [1,2]. With landmark clinical trials [3-6] demonstrating the efficacy of the latter drugs, there has been a paradigm shift in initiating and sequencing optimal anti–heart failure therapy and a concomitant reevaluation of the evidence base that guides conventional heart failure management. The American Heart Association heart failure guideline supports the rapid titration of guideline-based therapies every 1-2 weeks, with the goal of achieving target doses [7]. However, the European Society of Cardiology does not contain a timeline for the initiation of guideline-based heart failure therapy [8]. Pharmacologically, it is rational to use a combination of drugs that target multiple pathological mechanisms, as there is potential synergism and better therapeutic outcomes. Theoretically, the quicker the right combinations are used, the more likely the beneficial effects will be experienced. However, rapid up-titration must be balanced with patient safety and tolerability.

Sacubitril and valsartan, β-blockers, mineralocorticoid receptor antagonists (MRAs), and SGLT2is are disease-modifying agents that are used as combination therapy and are now regarded as a foundational therapy for HFrEF [1,2]. The conventional approach to initiating and optimizing HFrEF therapy reflects the sequence in which drugs were developed and trialed over the past 40 years [1]. Clinicians initiate therapy with an angiotensin-converting enzyme inhibitor or angiotensin receptor blocker, followed by a β-blocker, then an MRA, then a neprilysin inhibitor, and, finally, an SGLT2i. Each drug required up-titration to the target dose, or the maximally tolerated dose, before initiating another class of drug, with a treatment period usually spanning >6 months [1].

This approach is limited by several clinical misconceptions and assumptions: first, the most efficacious anti–heart failure therapies were developed first. The counterargument is exemplified by the fact that digitalis has been used in clinical practice for 200 years, yet it is no longer considered a key therapeutic agent. Second, drug efficacy is only achieved at maximum target doses [1]. However, studies have demonstrated morbidity and mortality reduction with the use of low-dose anti–heart failure therapy in HFrEF, with maximum target doses adding only a smaller clinical benefit. Third, the efficacy and safety of individual anti–heart failure drug class were assessed in clinical trials that required patients to be receiving all background therapy at target doses. However, most trials were conducted with patients receiving subtarget doses of anti–heart failure therapy [2].

Initiating drugs over a 6-month period is undesirable for several reasons. Practically, target doses of anti–heart failure therapy are infrequently achieved due to patient factors (nonadherence, perceived adverse effects, and costs), drug factors (adverse effects and frequency of dosing), physician factors (clinical inertia and limited time), inadequate patient follow-up, and a lack of perceived benefit of higher drug doses. Furthermore, there is a strong evidence base suggesting that the use of each of the foundation drugs has demonstrated morbidity and mortality reduction within 30 days of therapy initiation [1,2,9].

The aim of this study is to determine if early addition of ARNIs, SGLT2is, β-blockers, and MRAs (within 4 weeks), when compared with the same therapies initiated slower (within 6 months), will reduce all-cause mortality and hospitalizations for heart failure in patients with stable HFrEF.

### Rationale

The use of ARNIs and SGLT2is in low- to middle-income country settings is limited by high cost and accessibility. Despite the strong body of evidence supporting the safety and efficacy of ARNIs and SGLT2is, these agents are not yet included in the South African Essential Drug List. ARNIs and SGLT2is are available to private funders, yet access is limited as costs are only partially reimbursed by medical insurance schemes. Furthermore, SGLT2is have not yet been approved for heart failure by the South African Health Products Regulatory Authority, and use within clinical practice is predominantly off-label. Donations by the pharmaceutical industry have enabled limited access to ARNIs and SGLT2is in the public sector.

The challenge in this regard is maximizing access and rationalizing the use of ARNIs and SGLT2is while canvassing for widespread cost-effective rollout in the public sector.

The data on ARNIs and SGLT2is from high-income country settings suggest that morbidity and mortality reduction is achieved early in the clinical presentation of heart failure [1,2]. There, however, remains a paucity of data on the safety and efficacy of these drugs in sub-Saharan Africa.

Within sub-Saharan Africa, the main driver of heart failure remains hypertension, with ischemic heart disease emerging as a key etiology. There, however, is a growing cohort of young patients with idiopathic dilated cardiomyopathy with HFrEF who are often suboptimally medically treated with minimum prospects of receiving heart transplants. In our opinion, such a group of patients would ideally benefit from ARNIs and SGLT2is. Data from key clinical trials have demonstrated the use of ARNIs as de novo therapy in patients presenting with heart failure [3,10,11]. The latter trials have argued against the use of angiotensin-converting enzyme inhibitors or angiotensin receptor blockers upfront with a transition phase to ARNI therapy, as there would be merely a delay in using the most effective therapy with a concomitant risk of sudden cardiac death.

The rapid initiation of anti–heart failure therapy described above seems rational in a high-income country setting where drug monitoring and assessment of adverse reactions are facilitated by the ease and rapidity of access to health care facilities [1,2]. However, in low- to middle-income country settings, the infrequent and irregular access to health care facilities limits instituting the rapid initiation algorithm for heart failure therapy. Furthermore, there is a lack of robust data and randomized controlled trials comparing rapid initiation versus a slower, phased initiation of heart failure therapy [1].

### Study Objectives

#### Primary Objective

The primary efficacy end point is the composite end point of all-cause mortality and hospitalization for heart failure.

#### Secondary Objectives

Secondary end points include death from cardiovascular causes (eg, cardiogenic shock, myocardial infarction, tachycardia, or brady-arrythmia), improvement in the 6-minute walk test, improvement in the New York Heart Association functional class, improvement in symptoms as per the Kansas City Cardiomyopathy Questionnaire, trans-esophageal echo-determined ejection fraction (chamber dilatation, left ventricular systolic function, global longitudinal strain, and speckle tracking), and N-terminal prohormone of brain natriuretic peptide levels. Adverse drug reactions will also be collected and analyzed.

### Definitions

#### Heart Failure

Heart failure is defined as a “clinical syndrome consisting of cardinal symptoms (eg, breathlessness, ankle swelling, and fatigue) that may be accompanied by signs (eg, elevated jugular venous pressure, pulmonary crackles, and peripheral edema). It is due to a structural and/or functional abnormality of the heart that results in elevated intracardiac pressures and/or inadequate cardiac output at rest and/or during exercise” [7].

#### Reduced Left Ventricular Ejection Fraction

Reduced left ventricular ejection fraction is defined as an ejection fraction ≤40%, that is, those with a significant reduction in left ventricular systolic function [8].

## Methods

### Proposed Novel and Rapid Sequencing

The proposed algorithm for initiation of anti–heart failure therapy comprises of 3 steps once clinical euvolemia has been achieved with diuresis [2].

Step 1 is the concurrent initiation of treatment with a β-blocker and an SGLT2i. β-Blockers are pertinent in the treatment of HFrEF, especially in the context of reducing sudden cardiac death. Evidence has demonstrated reduced hospitalization for heart failure with the use of SGLT2is. The early diuretic effect of SGLT2is may attenuate the early risk of worsening heart failure when β-blockers are initiated [2].

Step 2 comprises of the addition of an ARNI (sacubitril and valsartan) within 1-2 weeks of step 1. The presence of hypotension (systolic blood pressure <100 mm Hg) warrants evaluation of blood response with an angiotensin receptor blocker before switching to an ARNI [2].

Step 3 is the addition of an MRA, within 1-2 weeks of step 2, depending on the serum potassium and the presence and degree of renal impairment. ARNIs and SGLT2i may improve renal function and potassium homeostasis, thereby permitting the use of MRAs. In patients with hypotension, MRAs may be used as step 2 [2].

The above algorithm represents a generic approach and may be individualized to specific scenarios. Patients with decompensated heart failure are a subgroup that require greater caution when initiating anti–heart failure therapy. β-Blocker therapy should only be initiated in the hospital after discontinuation of intravenous therapy for several days and the patient is clinically euvolemic, defined as the absence of rales and ascites and the presence of minimal peripheral edema [2].

**Figure 1:**
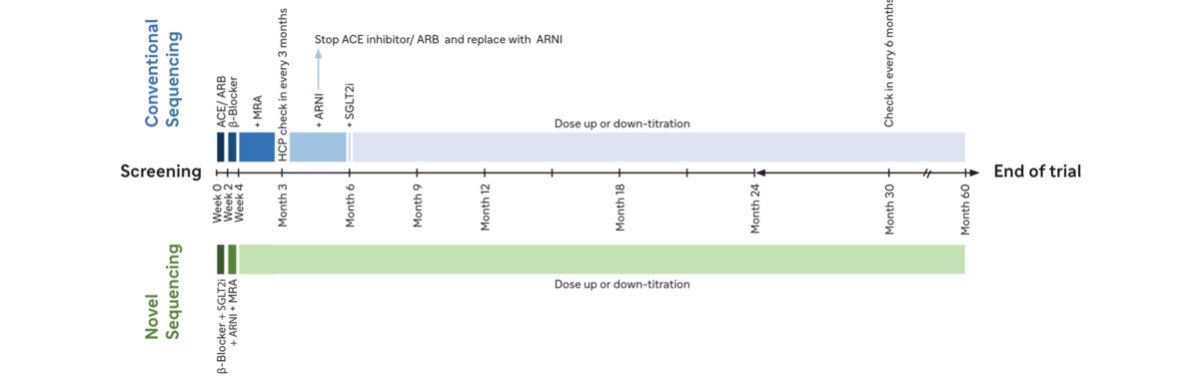
Study flow diagram. ACE: angiotensin-converting enzyme; ARB: angiotensin receptor blockers; ARNI: angiotensin receptor-neprilysin inhibitors; HCP: health care professional; MRA: mineralocorticoid receptor antagonists; SGLT2i: sodium/glucose cotransporter 2 inhibitors.

In the appropriate patient, treatment with all 4 foundational treatments may be achieved within 4 weeks, with subsequent dose up-titration. This strategy increases the probability that highly effective therapies will be implemented in a manner that reduces mortality and hospitalizations and enhances the tolerability of subsequently administered treatments [1]. Figure 1 shows the proposed study flow diagram, which contains the novel and conventional sequencing approaches. A key difference is the rate at which therapies are added, with the novel sequence arm having patients on all the efficacious therapies initiated within 4 weeks.

#### Study Design

This is a prospective, double-arm, randomized, assessor-blinded, and pragmatic clinical trial. The study will take place at a single site, that is, Tygerberg Hospital, Western Cape, South Africa.

#### Selection of Patients

Study participants will be adults with idiopathic dilated cardiomyopathy who consent to participate in the study. Patients will be free to withdraw at any time. The recruitment period will be 1 year.

Study participants will be randomized to the study arms by using a computer-generated randomization sequence.

Inclusion, exclusion, and withdrawal criteria are listed in Textbox 1.

**Selection criteria (inclusion, exclusion, and withdrawal criteria).**

**Inclusion criteria**

- All patients aged <18 or >50 years who fulfill the clinical criteria for idiopathic dilated cardiomyopathy with reduced ejection fraction
- New York Heart Association Functional class II-IV

**Exclusion criteria**

- Aged >18 years or <50 years
- Unable or unwilling to provide informed consent to undergo any of the recommended investigations or to participate in the study
- Acute coronary syndrome, bypass graft surgery or other major cardiac surgery, stroke, or transient ischemic attack ≤90 days from visit 1
- Acute decompensated heart failure
- Systolic blood pressure ≥180 mm Hg or <100 mm Hg
- Estimated glomerular filtration rate <30 mL/min/1.73 m^2^
- Serum potassium level >5.4 mmol/L
- History of angioedema

**Withdrawal criteria**

- Patient decision to leave the study
- Adverse drug reactions

#### Selection of Investigators

This study will be conducted at a tertiary-level public hospital’s department of cardiology. Investigators will be the authors of this publication and other staff based at Tygerberg Hospital.

### Statistical Considerations

#### Sample Size

We determined that a target number of 248 primary outcome events would provide a power of 80% to detect a 30% lower relative risk of the primary outcome in the novel sequencing arm than in the conventional sequencing arm at a 2-sided α level of .05. Assuming an incidence of the primary outcome of at least 50% over 5 years in the conventional sequencing arm and a dropout of 10% per arm, we established a planned enrolment of 584 patients (292 per study arm).

#### Analysis Population

The analysis population will include all study participants included in the study. This is an intention-to-treat analysis and even participants who have withdrawn, or dropped out, from the study after randomization will have their data analyzed.

#### Statistical Methods

Patient demographic and clinical profiles will be described using the statistical software SAS (version 9.4; SAS Institute). Continuous variables will be expressed as an absolute number, with associated percentage, mean, SD, median, and range where applicable. Clinical assessment of response to heart failure therapy will be performed using the Kansas City Cardiomyopathy Questionnaire, New York Heart Association functional classification, 6-minute walk distance, and hospital admission for heart failure. Objective measures of therapy response will be ascertained by serial N-terminal prohormone of brain natriuretic peptide levels and 2D echocardiography. Echocardiographic features of response to therapy will be characterized according to left ventricular ejection fraction as determined by the Simpson biplane method, global longitudinal strain, and speckle tracking.

Associations between outcomes and indicator variables will be explored through logistic regression. Where appropriate, indicator variables and specific outcomes will be investigated individually through a univariate analysis. Where significant, variables will be entered into and controlled for in a Cox-proportional hazards regression model. Adjustment for confounding will be performed by a competing risks analysis and propensity score matching (Table 1).

**Table 1:** Statistical methods.

Outcome measures		Hypothesis	Measure	Time points	Analysis methods
**Primary** **end points**		The intervention will reduce the outcome from baseline to 5 years.	—^a^	—	—
	All-cause mortality	Rapid sequencing of anti–heart failure therapy will reduce all-cause mortality.	All-cause mortality (binary)	End of trial	Cox proportional hazards regression
	Hospitalization	Rapid sequencing of anti–heart failure therapy will reduce all-cause mortality.	Hospitalized for heart failure (binary)	End of trial	Cox proportional hazards regression
**Secondary end points**					
	Deaths (from cardiovascular causes)	Intervention will reduce cardiovascular-related deaths	Cardiovascular mortality (binary)	End of trial	Chi-square test
	6-minute walk test	Improvement in distance walked in 6-minutes	Distance in meters (continuous)	Baseline and end of trial	✓
	NYHA^b^ functional class	Improvement in dyspnea	Functional class 1, 2, 3, and 4 (ordinal)	Baseline and end of trial	✓
	KCCQ^c^	Improvement in symptoms of heart failure	Questionnaire (ordinal)	Baseline and end of trial	✓
	Trans-esophageal echo	Improvement in ejection fraction	Ejection fraction measured in percentage (%; continuous)	Baseline and end of trial	✓
	NT-pro^d^ and BNP^e^ levels	Reduction in levels	Concentration units (pg/mL; continuous)	Baseline and end of trial	✓

^a^Not applicable.

^b^NYHA: New York Heart Association.

^c^KCCQ: Kansas City Cardiomyopathy Questionnaire.

^d^NT-pro: N-terminal prohormone.

^e^BNP: brain natriuretic peptide.

### Ethical Considerations

This study will be conducted in accordance with the principles laid down by the 18th World Medical Assembly [12]. Study participants’ data will be deidentified of names and surnames and identifying information. An application for ethical approval will be submitted to the Stellenbosch University Research and Ethics Committee. The study will be performed in accordance with local regulations, including local data protection regulations and requisite hospital and Provincial Department of Health Approval. Posttrial access and care will be provided to all study participants, throughout their lifespan.

## Results

We are currently in the process of generating funding. If funding is obtained, we will apply for ethical clearance and requisite permissions.

## Discussion

### Principal Findings

This study aims to determine if rapid sequencing of anti–heart failure drugs with proven morbidity and mortality benefits would be superior to slower titration of these therapies.

We chose a composite end point to reduce the sample size, as a larger sample size would result in greater costs and reduce the feasibility of the study. Given the high mortality and frequent hospitalizations for heart failure, we included both events in the combined primary efficacy endpoint.

Intuitively, it makes sense to ensure that the most efficacious heart failure therapies are initiated as soon as possible. Given that the therapies have varied modes of action, we hypothesize that if they are started together as soon as possible, they would have greater benefits than starting them later. Combination therapies are likely to target different pathophysiological aspects of heart failure and thus improve clinical outcomes. Issues with rapid initiation may potentially include hypotension and other adverse effects. We will guard against hypotensive events by starting with the lowest possible dose and stopping the drug if the patient becomes hemodynamically unstable. Furthermore, study participants are required to be euvolemic before rapid sequencing so that we do not worsen patients’ heart failure and thus limit hemodynamic instability and decompensated heart failure. It is a standard clinical practice to ensure euvolemia before titration of anti–heart failure therapies. Euvolemia is usually achieved by loop diuretics [8].

We chose a relatively young group of patients so that underlying issues such as ischemic heart disease are less probable and would not serve as confounders. Furthermore, this group of patients may benefit from the increased life expectancy as compared with the older group.

The public health sector in South Africa has resource constraints. Often, there are not enough clinicians to adequately manage the patients. Thus, doing trials in this setting is challenging. However, given the proven safety and efficacy of ARNIs, SGLT2is, and MRAs, and their widespread use in high-income countries, we envisage limited problems. Furthermore, making these novel therapies available to a vulnerable population would serve as a motivation for clinicians who work in this setting. The setting is a teaching and training facility, and having exposure to these novel therapies will grow the expertise and skills of health care practitioners. Furthermore, we envisage reducing the complexity of the study by using a pragmatic study design so that we can integrate the trial into routine clinical practice in the department of cardiology. This will reduce the administration burden while also resulting in the collection of safety, efficacy, and tolerability data. We feel that the trial would not add to the administrative burden of the clinicians, as we will not ask them to do any work beyond what they usually do in the clinic. Furthermore, a separate designated individual will collect and analyze the data, so that clinicians will not be burdened with this task and not impinge on their demanding clinical work.

Posttrial access and posttrial care are contentious issues [13]. In this study, we plan to provide both posttrial access and posttrial care because it is, in our opinion, ethically justified. Given that the newer therapies are efficacious, safe, and tolerable, it would be unkind to stop the therapies after the trial is completed, as the study participants would not be able to procure the therapy privately because of their limited financial resources. The provision of posttrial care would also increase the cost of the study, but we will continue to provide posttrial care in our cardiology clinic at no cost to the study participants.

We hope to acquire funding from the manufacturers of ARNIs, SGLT2is, β-blockers, and MRAs, as the data generated from this study have the potential to inform clinical practice guidelines. Given the high costs of doing such trials, we hope companies will assist if they are only providing the drug and not covering the other clinical trial costs like investigator fees, protocol development, manuscript writing, and statistical analysis, and so on. The study has dual objectives of generating of evidence while also providing patients with access to highly efficacious and premium therapies for heart failure.

Given that both study groups will receive the same therapies, we do not pitch drugs head-to-head, as we feel that the combination of drugs with proven mortality and morbidity data would be most likely to have synergistic effects and further reduce morbidity and mortality. This approach is also more likely to generate funding as all companies can contribute to financing the study, not with the risk of their products being negatively affected by head-to-head trials that show one drug being superior to another drug.

We have tried to create a “win-win” situation by generating data to inform clinical practice guidelines and providing access to novel therapies in resource-limited settings. The research can help with master’s and PhD degrees, given the rich dataset generated.

### Limitations

The study is proposed to use one study site in the Western Cape of South Africa, which may not be reflective of the demographics of the South African population. Due to the nature of the study and means of intervention, implementation of a placebo-controlled arm will not be possible but is nonetheless cited as a limitation.

### Conclusions

Recent additions to the armamentarium of anti–heart failure therapies such as ARNIs and SGLT2is have demonstrated morbidity and mortality benefits. Earlier use of such therapy within patients with stable HFrEF may impact patient outcomes. This study will provide important data to support evidence-based medicine and will help inform clinical practice guidelines on the optimal rate of sequencing of anti–heart failure therapies.

## Abbreviations

ARNI: angiotensin receptor-neprilysin inhibitor
HFrEF: heart failure with reduced ejection fraction
MRA: mineralocorticoid receptor antagonist
SGLT2i: sodium/glucose cotransporter 2 inhibitor

## Data Availability

The dataset will be made available upon written request.
